# An interim review of the epidemiological characteristics of 2019 novel coronavirus

**DOI:** 10.4178/epih.e2020006

**Published:** 2020-02-06

**Authors:** Sukhyun Ryu, Byung Chul Chun, Korean Society of Epidemiology 2019-nCoV Task Force Team

**Affiliations:** 1 Department of Preventive Medicine, Konyang University College of Medicine, Daejeon, Korea; 2 Department of Preventive Medicine, Korea University College of Medicine, Seoul, Korea

**Keywords:** Coronavirus, Epidemiology, Characteristics, Public health

## Abstract

**OBJECTIVES:**

The 2019 novel coronavirus (2019-nCoV) from Wuhan, China is currently recognized as a public health emergency of global concern.

**METHODS:**

We reviewed the currently available literature to provide up-to-date guidance on control measures to be implemented by public health authorities.

**RESULTS:**

Some of the epidemiological characteristics of 2019-nCoV have been identified. However, there remain considerable uncertainties, which should be considered when providing guidance to public health authorities on control measures.

**CONCLUSIONS:**

Additional studies incorporating more detailed information from confirmed cases would be valuable.

## INTRODUCTION

**Figure 1.**
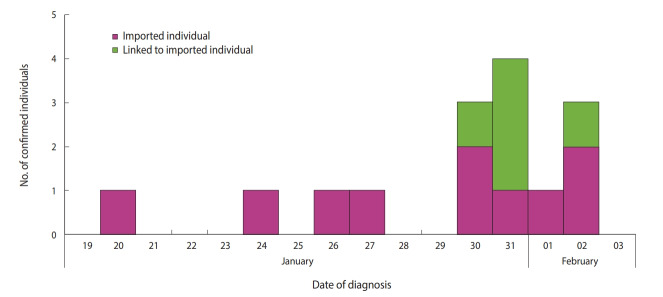
Timeline of individuals with laboratory-confirmed 2019 novel coronavirus infections in Korea, as of February 3, 2020.

Several clusters of patients with pneumonia of unknown etiology in Wuhan, Hubei Province, China were reported to the Chinese health authorities starting on December 8, 2019, and most of these cases were epidemiologically linked to a local fish and animal market [1,2]. The pathogenic agent responsible for these clusters of pneumonia was identified as a 2019 novel coronavirus (2019-nCoV) [1]. At the very beginning of the 2019-nCoV outbreak in China, much remained unknown, except for the fact that it was transmitted by direct exposure at the market [3]. However, person-to-person transmission of 2019-nCoV has been confirmed [4], and asymptomatic individuals have been identified as potential sources of infection [5]. The number of identified cases has been steadily growing, and as of February 3, a total of 14,557 cases had been reported globally (14,411 in China and 146 in 22 other countries) [6]. Since the first laboratory-confirmed case was identified on January 20, 2020 in Korea, the number of reported cases grew to 15 as of February 3, 2020 (Figure 1 and Table 1) [7].

There remain considerable knowledge gaps on 2019-nCoV; therefore, the public health authorities in countries with any likelihood of experiencing imported cases of 2019-nCoV should update the case definition to reflect newly updated epidemiological data. Herein, we present a review of the literature on the epidemiological characteristics of human infections with 2019-nCoV to provide an interim summary to public health authorities.

## MATERIALS AND METHODS

We searched the literature for studies reporting epidemiological characteristics of 2019-nCoV, including the reproductive number, incubation period, serial interval, infectious period, generation time, latent period, and the fatality rate of hospitalized cases. The Korean Society of Epidemiology 2019-nCoV Task Force Team (KSE 2019-nCoV TFT) searched for peer-reviewed articles published from December 8, 2019 to February 1, 2020. Articles were eligible for inclusion if they reported any epidemiological characteristics of 2019-nCoV.

### Ethics statement

The ethical approval or individual consent was not applicable.

## RESULTS

Six articles were identified and included in this review (Table 2) [2,8-12] . Four relevant studies estimated the reproductive number [8,10-12]. A study of confirmed cases from Wuhan, China estimated the reproductive number to be 1.9 (95% confidence interval [CI], 1.3 to 3.2) [10]. Three other studies estimated the mean reproductive number as 0.3, 2.2, and 2.68, respectively [8,11,12]. A study reported the mean incubation period to be 6.1 days (95% CI, 3.8 to 9.7), and the mean serial interval to be 7.7 days (95% CI, 4.9 to 13.0) [10]. Two studies predicted the mean doubling time to be between 6.4 days and 8.7 days [10,11]. Three studies estimated the fatality rate of hospitalized cases as 14-15% [2,8,9]. We could not identify any studies that reported the infectious and latent periods.

## DISCUSSION

We reviewed the epidemiological characteristics of 2019-nCoV. The estimated reproductive number of 0.3 was obtained from a small number of infected persons with imperfect information in the very early stages of the outbreak [8]; therefore the reproductive number of 2019-nCoV is likely to be similar to that of the 2002/2003 severe acute respiratory syndrome (SARS) coronavirus during the pre-intervention period (range, 2 to 3) and that of the 2009 pandemic A/H1N1 influenza virus in the United States (range, 1.3 to 1.7) [13-15]. The incubation period is likely similar to that of the SARS coronavirus, but with a wider confidence interval (mean, 4.8 days; 95% CI, 4.2 to 5.5) [16]. Furthermore, it is longer than that of the 2009 pandemic A/H1N1 influenza virus (median incubation period, 1.4 days; 95% CI, 1.0 to 1.8) [17]. Therefore, the evidence reviewed above shows that the current control measures for 2019-nCoV, including a quarantine and observation period of 14 days for suspected cases, can be considered appropriate [10]. The generation time and serial interval of 2019-nCoV are longer than those of the 2009 pandemic A/H1N1 influenza virus (median generation time, 2.7 days; 95% CI, 2.0 to 3.5; and mean serial interval: range, 2.6 to 3.2) [14,18]. However, the mean serial interval of 2019-nCoV is similar to that of the SARS coronavirus (mean, 8.4 days; standard deviation, 3.8) [19]. The overall case fatality rate of 2019-nCoV was estimated by international experts to range from 3% to 14% [15,20], and it is more likely to cause infection in older age groups with commodities [2].

Epidemiological parameters are usually obtained from a consecutive timeline of the number of reported cases and contact-tracing data. However, most of the studies included in our review made estimates using data obtained from the early stages of the outbreak in Wuhan, in which the reporting of confirmed cases may have been incomplete. Furthermore, differences in the simulation methodology used in various scenarios may result in a wide spectrum of estimated values [21]. Therefore, caution is needed when interpreting these reported results.

The number of confirmed cases is increasing in China and in other countries, including Korea. Furthermore, the likelihood of local transmission is increasing as a result of cases entering from China.

In light of a report describing a case of human-to-human transmission in the asymptomatic period [5], it is necessary to consider updating the case definition for surveillance; however, more detailed studies presenting evidence on the epidemiological nature, clinical presentation, and pathogenesis of 2019-nCoV are necessary to provide information for public health decision-making.

## Figures and Tables

**Table 1.** List of confirmed cases of 2019 novel coronavirus infection in Korea, as of February 3, 2020

Case No.	Age (yr)	Sex	Nationality	Date of entry to Korea	Suspected infection of place or origin
#1	35	Female	Chinese	Jan 19, 2020	Wuhan, China
#2	55	Male	Korean	Jan 22, 2020	Wuhan, China
#3	54	Male	Korean	Jan 20, 2020	Wuhan, China
#4	55	Male	Korean	Jan 20, 2020	Wuhan, China
#5	33	Male	Korean	Jan 24. 2020	Wuhan, China
#6	55	Male	Korean	-	Case #3
#7	28	Male	Korean	Jan 23, 2020	Wuhan, China
#8	62	Female	Korean	Jan 23, 2020	Wuhan, China
#9	28	Female	Korean	-	Case #5
#10	54	Female	Korean	-	Case #6
#11	25	Male	Korean	-	Case #6
#12	48	Male	Chinese	Jan 19, 2020	Osaka, Japan
#13	28	Male	Korean	Jan 31, 2020	Wuhan, China
#14	40	Female	Chinese	-	Case #12
#15	43	Male	Korean	Jan 20, 2020	Wuhan, China

**Table 2.** Summary of reviews included in the study

Study	Study setting	Reproductive (n)	Incubation period (d)	Serial interval (d)	Infectious period	Doubling time (d)	Latent period	Fatality rate among hospitalized cases (%)
Wu et al. [8]	Publicly-available data in China as at Jan 22, 2020	0.30 (95% CI: 0.17, 0.44)	NA	NA	NA	NA	NA	14.0 (95% CI: 3.9, 32.0)
Huang et al. [9]	41 confirmed cases admitted to a designated hospital in Wuhan by Jan 2, 2020	NA	NA	NA	NA	NA	NA	15.0
Chen et al. [2]	99 confirmed cases admitted in Wuhan Jinyintan Hosptial between Jan 1 and Jan 20, 2020	NA	NA	NA	NA	NA	NA	14.6
Li et al. [10]	425 confirmed cases in Wuhan as at Jan 22, 2020	1.9 (95% CI: 1.3, 3.2)	6.1 (95% CI: 3.8, 9.7)	7.7 (95% CI: 4.9, 13.0)	NA	8.7 (95% CI: 4.8, 17.0)	NA	NA
Riou et al. [12]	Modelling study	2.2 (90% CI: 1.4, 3.8)	NA	NA	NA	NA	NA	NA
Wu et al. [11]	Modeling study	2.68 (95% CrI: 2.47, 2.86)	NA	NA	NA	6.4 (95% CrI: 5.8, 7.1)	NA	NA

CI, confidence interval; Crl, credible interval; NA, not available.

## SUPPLEMENTARY MATERIALS

Korean version is available at http://www.e-epih.org/.
