# Supplementary material for: An interim review of the epidemiological characteristics of 2019 novel coronavirus
Source: Epidemiol Health. 2020 Feb 6;42:e2020006. doi: 10.4178/epih.e2020006 (PMC7011107; doi:10.4178/epih.e2020006)
Supplement: Supplementary file 1 [file epih-42-e2020006-suppl.pdf]

Volume: 42, Article ID: e2020006
https://doi.org/10.4178/epih.e2020006

BRIEF COMMUNICATION

An interim review of the epidemiological characteristics of 2019 novel coronavirus

Sukhyun Ryu[1], Byung Chul Chun[2]; Korean Society of Epidemiology 2019-nCoV Task Force Team*

[1]*Department of Preventive Medicine, Konyang University College of Medicine, Daejeon, Korea;* [2]*Department of Preventive Medicine, Korea University College of Medicine, Seoul, Korea*

초록

목적: 신종코로나바이러스 (2019-nCoV) 유행의 세계적인 우려 속에 세계보건기구는 공중보건 비상사태를 선포하였다.

방법: 현재 공중보건 당국의 관리방안 제시 및 평가하기 위해 문헌 리뷰를 시행하였다.

결과: 일부 역학적 특성들이 확인되었지만, 유행 관리방안의 최신화 된 지침을 제공하기엔 여전히 많은 것들이 불확실하다.

결론: 확진 감염자의 자세한 정보를 바탕으로 한 추가 역학 연구들이 필요하다.

도입

2019년 12월 8일부터 중국 후베이성 우한에서 병인이 알려지지 않은 폐렴 환자 군집이 중국 보건당국에 보고되었으며, 이들 중 대부분은 역학적으로 현지 어류 및 동물 시장과 관련이 있었다 [1, 2]. 이러한 환자 군집들의

Volume: 42, Article ID: e2020006
https://doi.org/10.4178/epih.e2020006

병원체는 신종코로나바이러스 (2019-nCoV)로 확인되었다 [1]. 중국에서 발생한 신종코로나바이러스 집단발병 초기에는 시장에 직접 노출되어 전파되었다는 사실 이외에는 알려진 것이 거의 없었다 [3]. 그러나 신종코로나바이러스의 사람 간 전파가 확인되었고 [4], 무증상기 전파 가능성도 제기되었다 [5].

2월 3일 현재 확진자의 수가 증가하여, 전 세계적으로 14,557건(중국 및 22개국에서 각각 14,411건, 146건)이 보고되었다 [6]. 한국에서는 2020년 1월 20일 첫 실험실 확진 사례가 확인된 이후, 2020년 2월 3일까지 15명의 확진자가 보고 되었다 (그림 1, 표 1) [7].

신종 코로나바이러스에 대한 특성은 아직도 알려져 있지 않은 부분이 많다. 따라서 신종 코로나바이러스의 유행 가능성이 있는 국가는 최신 역학 자료에 따라 사례 정의 및 관리방안 등을 최신화 하여야 할 필요가 있다. 우리는 신종 코로나바이러스(2019-nCoV)에 의한 인체감염의 역학적 특성에 관한 문헌들을 검토하고, 그 중간 요약내용을 보건당국에 제공하기 위해 본 연구를 진행하였다.

재료와 방법

한국역학회의 신종코로나바이러스 대책위원회는 2019년 12월 8일부터 2020년 2월 1일까지 발행된 감염재생산수, 잠복기, 연속간격, 감염기간, 세대기, 잠재기, 입원 환자에 대한 치사율 등 역학적 특성이 포함된 문헌들을 검색하였다.

결과

전체 6개의 논문이 확인되었다 (표 2) [2, 8-12]. 4개의 관련 문헌에서는 감염재생산지수를 추정했다 [8, 10-12]. 중국 우한의 확진 사례를 이용한 연구에서 감염재생산수의 추정치는 1.9였다 (95% 신뢰 구간: 1.3–3.2) [10]. 3개의 연구에서 평균 감염재생산수를 0.3, 2.2 그리고 2.68로

추정하였다 [8, 11, 12]. 또 다른 한 연구에서 잠복기(평균, 6.1일, 95% 신뢰구간, 3.8-9.7일) 및 연속 간격 (평균, 7.7일, 95% 신뢰구간, 4.9-13일)을 추정하였다 [10]. 2개의 연구에서 세대기는 6.4-8.7일 사이라고 예측했고 [10,11], 3개 연구에서는 병원 치명률이 14-15% 사이인 것으로 추정했다 [2,8,9]. 전염력과 잠재기가 보고된 연구는 확인할 수 없었다.

고찰

우리는 신종코로나바이러스의 역학적 특성들을 검토했다. 감염재생산수 0.3은 소수의 감염자와 집단발병 초기의 불완전한 정보로 얻은 수치였다 [8]; 그러므로 신종코로나바이러스의 감염재생산수는 2002/2003년 사스 코로나이러스 (범위, 2-3)과 미국에서 측정된 2009년 인플루엔자 A/H1N1 바이러스와 유사할 가능성이 높다 (범위: 1.3-1.7) [13-15]. 잠복기는 사스 코로나바이러스 (평균 4.8일, 95% 신뢰구간:4.2-5.5)와 비슷하지만, 신뢰 구간은 신종 코로나바이러스가 더 넓다 [16]. 또한 2009년 인플루엔자 A/H1N1 바이러스 (평균 잠복기, 1.4일, 95% 신뢰구간, 1.0-1.8일)보다 길다 [16, 17]. 따라서 신종코로나바이러스 의심환자에 대해 14일간의 격리 및 관찰 기간은 역학적으로 적절한 것으로 판단된다 [10].

세대기 및 연속 간격은 2009년 인플루엔자 A/H1N1 바이러스 (평균 세대기 , 2.7일, 95% 신뢰구간, 2.0-3.5 및 평균 연속 간격, 범위 2.6-3.2)보다 길다 [14, 18]. 그러나 평균 연속 간격은 사스 코로나바이러스 (평균, 8.4; 표준편차 3.8일)와 비교적 유사하다 [19].

국제 전문가들은 신종 코로나바이러스의 전체 치명률 범위를 3-14%로 추정했으며 [15, 20], 다른 질환이 동반된 노년층의 치명률이 높음을 보고하였다 [2]. 우리가 선정하여 검토한 문헌에서 추정된 값들은 우한에서 발생한 집단발병 초기단계에서 얻은 자료를 토대로 했기 때문에, 확진 사례에 대한 보고가 불완전할 수 있다. 또한, 여러 다른 상황에서의

모의실험 방법을 통한 연구는 광범위한 범위를 갖는 결과값을 추정하고 있다 [21]. 따라서, 이들 보고된 결과 값에 대한 해석은 신중할 필요가 있다.

중국을 비롯하여 확진 판례가 한국을 포함한 다른 나라들에서 증가하고 있다. 또한, 중국으로부터 한국으로 입국 사례들이 지속됨에 따라 지역사회 전파의 가능성 또한 증가하고 있다. 무증상 기간에 사람 간 전파 사례가 보고됨에 따라 [5], 감시체계 개선을 위한 사례 정의의 변경을 고려할 필요가 있다. 공공 보건 의사결정에 대한 정보를 제공하기 위해 신종 코로나바이러스의 발생기전뿐만 아니라 임상 증상 등의 다양하고 지속적인 역학 연구들이 필요하다.

이해의 상충

저자들은 이 연구와 관련하여 이해관계의 상충이 없음.

감사의 말씀

한국역학회 신종 코로나바이러스 감염증 대책위원회 전원(알파벳 순)
Byung Chul Chun (Department of Preventive Medicine, Korea University College of Medicine, Seoul); Dong Hyun Kim (Department of Social and Preventive Medicine, Hallym University College of Medicine, Chuncheon); Hae-Kwan Cheong (Sungkyunkwan University School of Medicine, Suwon); Heeyoung Lee (Center for Preventive Medicine and Public Health, Seoul National University Bundang Hosptial, Seongnam); Hyunjin Son (Busan Center for Infectious Disease Control and Prevention, Busan National University Hospital);
Ji-Hyuk Park (Department of Preventive Medicine, Dongguk University, Gyeongju), Jong-Hun Kim (Sungkyunkwan University School of Medicine, Suwon); Kwan Lee (Department of Preventive Medicine, Dongguk University, Gyeongju); Kwang-Pil Ko (Department of Preventive Medicine, Gachon

University, College of Medicine, Incheon); Sukhyun Ryu (Department of Preventive Medicine, Konyang University, Daejeon); Sung-il Cho (Graduate School of Public Health, Seoul National University, Seoul); Young June Choe (Department of Social and Preventive Medicine, Hallym University College of Medicine, Chuncheon).

참고문헌

1. Zhu N, Zhang D, Wang W, Li X, Yang B, Song J, et al. A Novel Coronavirus from Patients with Pneumonia in China, 2019. New England Journal of Medicine. 2020.
2. Chen N, Zhou M, Dong X, Qu J, Gong F, Han Y, et al. Epidemiological and clinical characteristics of 99 cases of 2019 novel coronavirus pneumonia in Wuhan, China: a descriptive study. The Lancet.
3. The Lancet. Emerging understandings of 2019-nCoV. The Lancet. 2020;395(10221):311.
4. Chan JF-W, Yuan S, Kok K-H, To KK-W, Chu H, Yang J, et al. A familial cluster of pneumonia associated with the 2019 novel coronavirus indicating person-to-person transmission: a study of a family cluster. The Lancet.
5. Rothe C, Schunk M, Sothmann P, Bretzel G, Froeschl G, Wallrauch C, et al. Transmission of 2019-nCoV Infection from an Asymptomatic Contact in Germany. New England Journal of Medicine. 2020.
6. World Health Organization. Novel Coronavirus (2019-nCoV) Situation Report: World Health Organization; 2020.
7. Korean Ministry of Health Welfare. 2019-nCoV [cited 2020 Feb 3]. Available from :http://ncov.mohw.go.kr/tcmBoardList.do?brdId=&brdGubun=&dataGubun=&ncvContSeq=&contSeq=&board_id= (Korean).

8. Wu P, Hao X, Lau EHY, Wong JY, Leung KSM, Wu JT, et al. Real-time tentative assessment of the epidemiological characteristics of novel coronavirus infections in Wuhan, China, as at 22 January 2020. Eurosurveillance. 2020;25(3):2000044.

9. Huang C, Wang Y, Li X, Ren L, Zhao J, Hu Y, et al. Clinical features of patients infected with 2019 novel coronavirus in Wuhan, China. The Lancet.

10. Li Q, Guan X, Wu P, Wang X, Zhou L, Tong Y, et al. Early Transmission Dynamics in Wuhan, China, of Novel Coronavirus–Infected Pneumonia. New England Journal of Medicine. 2020.

11. Wu JT, Leung K, Leung GM. Nowcasting and forecasting the potential domestic and international spread of the 2019-nCoV outbreak originating in Wuhan, China: a modelling study. The Lancet.

12. Riou J, Althaus CL. Pattern of early human-to-human transmission of Wuhan 2019 novel coronavirus (2019-nCoV), December 2019 to January 2020. Eurosurveillance. 2020;25(4):2000058.

13. Wallinga J, Teunis P. Different Epidemic Curves for Severe Acute Respiratory Syndrome Reveal Similar Impacts of Control Measures. American Journal of Epidemiology. 2004;160(6):509-16.

14. Yang Y, Sugimoto JD, Halloran ME, Basta NE, Chao DL, Matrajt L, et al. The transmissibility and control of pandemic influenza A (H1N1) virus. Science. 2009 Oct 30;326(5953):729-33.

15. European Centre for Disease Prevention and Control (ECDC). Risk assessment: Outbreak of acute respiratory syndrome associated with a novel coronavirus, China: first local transmission in the EU/EEA − third update; 2020.

16. Wong TW, Tam W. Estimating SARS incubation period. Emerg Infect Dis. 2004 Aug;10(8):1503-4; author reply 4.

17. Lessler J, Reich NG, Cummings DAT. Outbreak of 2009 Pandemic Influenza A (H1N1) at a New York City School. New England Journal of Medicine. 2009;361(27):2628-36.

18. Balcan D, Hu H, Goncalves B, Bajardi P, Poletto C, Ramasco JJ, et al. Seasonal transmission potential and activity peaks of the new influenza A(H1N1): a Monte Carlo likelihood analysis based on human mobility. BMC Med. 2009;7:45-.

19. Lipsitch M, Cohen T, Cooper B, Robins JM, Ma S, James L, et al. Transmission dynamics and control of severe acute respiratory syndrome. Science. 2003 Jun 20;300(5627):1966-70.

20. Wang C, Horby PW, Hayden FG, Gao GF. A novel coronavirus outbreak of global health concern. The Lancet.

21. De Angelis D, Presanis AM, Birrell PJ, Tomba GS, House T. Four key challenges in infectious disease modelling using data from multiple sources. Epidemics. 2015 Mar;10:83-7.
